# Using text and charts to provide social norm feedback to general practices with high overall and high broad-spectrum antibiotic prescribing: a series of national randomised controlled trials

**DOI:** 10.1186/s13063-022-06373-y

**Published:** 2022-06-18

**Authors:** Natalie Gold, Anna Sallis, Ayoub Saei, Rohan Arambepola, Robin Watson, Sarah Bowen, Matija Franklin, Tim Chadborn

**Affiliations:** 1 Public Health England, Wellington House, 133-155 Waterloo Road, London, SE1 8UG UK; 2 Centre for Philosophy of the Natural and Social Science, London School of Economics and Political Science, Houghton Street, London, WC2A 2AE UK; 3 Behavioural Practice, Kantar Public, Millbank, Westminster London, SW1P 3JA UK; 4 Johns Hopkins Bloomberg School of Public Health, 615 N Wolfe St, Baltimore, MD 21205 USA; 5 Department of Psychology, University of Warwick, University Road, Coventry, CV4 7AL UK; 6 School of Economics, Sir Clive Granger Building, University Park, Nottingham, NG7 2RD UK; 7 Department of Experimental Psychology, University College London, 26 Bedford Way, London, WC1H OAP UK

**Keywords:** Antibiotics, Antimicrobial resistance, Behavioural intervention, Broad-spectrum prescribing, Data visualisation, Feedback, Messenger effect, Prescribing rates, Primary care, Social norms

## Abstract

**Background:**

Sending a social norms feedback letter to general practitioners who are high prescribers of antibiotics has been shown to reduce antibiotic prescribing. The 2017-9 Quality Premium for primary care in England sets a target for broad-spectrum prescribing, which should be at or below 10% of total antibiotic prescribing. We tested a social norm feedback letter that targeted broad-spectrum prescribing and the addition of a chart to a text-only letter that targeted overall prescribing.

**Methods:**

We conducted three 2-armed randomised controlled trials, on different groups of practices: Trial A compared a broad-spectrum message and chart to the standard-practice overall prescribing letter (practices whose percentage of broad-spectrum prescribing was above 10% and who had relatively high overall prescribing). Trial C compared a broad-spectrum message and a chart to a no-letter control (practices whose percentage of broad-spectrum prescribing was above 10% and who had relatively moderate overall prescribing). Trial B compared an overall-prescribing message with a chart to the standard practice overall letter (practices whose percentage of broad-spectrum prescribing was below 10% but who had relatively high overall prescribing). Letters were posted to general practitioners, timed to be received on 1 November 2018. The primary outcomes were practices’ percentage of broad-spectrum prescribing (trials A and C) and overall antibiotic prescribing (trial B) each month from November 2018 to April 2019 (all weighted by the number and characteristics of patients registered in the practice).

**Results:**

We randomly assigned 1909 practices; 58 closed or merged during the trial, leaving 1851 practices: 385 in trial A, 674 in trial C, and 792 in trial B. AR(1) models showed that there were no statistically significant differences in our primary outcome measures: trial A *β* = − .199, *p* = .13; trial C *β* = .006, *p* = .95; trial B *β* = − .0021, *p* = .81. In all three trials, there were statistically significant time trends, showing that overall antibiotic prescribing and total broad-spectrum prescribing were decreasing.

**Conclusion:**

Our broad-spectrum feedback letters had no effect on broad-spectrum prescribing; adding a bar chart to a text-only letter had no effect on overall antibiotic prescribing. Broad-spectrum and overall prescribing were both decreasing over time.

**Trial registration:**

ClinicalTrials.gov NCT03862794. March 5, 2019.

**Supplementary Information:**

The online version contains supplementary material available at 10.1186/s13063-022-06373-y.

## Introduction

Antimicrobial resistance (AMR) occurs because, over time, bacteria evolve to become resistant to antibiotics. At present, 700,000 people die of resistant infections every year, and this could rise to 10 million lives a year by 2050 [1]. In the UK, approximately 80% of antibiotics that are prescribed are prescribed in primary care [2]. AMR is accelerated by ‘inappropriate’ use of antibiotics, the prescribing of antibiotics when they are not clinically indicated and will have zero or marginal benefit. Modelling of primary care antibiotic prescribing shows that up to 23.1% of that prescribing is inappropriate and that all practices have the potential to make some reduction [3].

Social norm feedback can decrease antibiotic prescribing in general practice. In September 2014, a randomised controlled trial (RCT) showed that a ‘social norm feedback letter’ from the Chief Medical Officer (CMO) of England to general practitioners (GPs) in the highest prescribing practices in England, telling them that the great majority (80%) of practices in their local area team prescribe fewer antibiotics than them, led to a 3.3% reduction in prescribing [4]. The CMO continued to send a letter to high prescribers every winter. An evaluation of the winter 2016/2017 letter using a Regression Discontinuity Design showed that it continued to be effective, leading to a decrease in prescribing of 3.69% [5].

Broad-spectrum antibiotic prescribing is a particularly important driver of AMR [6]. The National Institute for Health and Care Excellence (NICE) recommends that narrow-spectrum antibiotics should generally be the first choice, both to preserve the efficacy of ‘last-line’ broad-spectrum agents and because broad-spectrum antibiotics can have worse side effects for the patient, since they can also kill commensal flora (non-harmful bacteria) leaving people susceptible to antibiotic-resistant harmful bacteria such as *Clostridium difficile* [7]. The 2017–2019 Quality Premium Scheme and the Clinical Commissioning Group (CCG) Improvement Assessment Framework set a target for broad-spectrum prescribing in primary care in England, which should be at or below 10% of total antibiotic prescribing [2]. However, social norm feedback letters in England so far had only targeted overall prescribing. Therefore, there was the potential to trial a letter with social norm feedback on broad-spectrum prescribing.

Other countries have also adopted a feedback letter as an intervention to reduce inappropriate antibiotic prescribing, including Australia [8], Northern Ireland [9, 10], and Canada [11, 12], with plans to introduce the intervention in France [13]. An RCT in Australia found that a social norm feedback letter had a very large effect, reducing prescribing by 12.3% compared to a control group who did not receive a letter [8]. There were two significant differences between the feedback in the Australian letter and the letter in England: the Australian letter provided information on the GP’s own prescribing compared to peers, not practice prescribing, and it contained a bar chart showing the GP’s prescribing compared to ‘your peers’. England does not at present collect data on individual GP prescribing, only practice-level data, but by adding a bar chart to the CMO letter, it would be possible to test whether the bar chart contributed to the large effect.

This trial took place in England, where the context was that the CMO had been sending an annual letter to high prescribing practices since 2014. Whether a practice received a letter as business as usual depended on its overall prescribing. Although the evidence is that all practices have the potential to reduce prescribing [3], we were constrained to only send letters to practices when that could be justified by their performance relative to national targets. The main aim was to test whether a social norm feedback letter would be effective at decreasing percentage broad-spectrum prescribing by practices who were not meeting the target; within this, practices varied in whether they would have qualified for a letter based on their overall prescribing. There was a group of practices who would be sent a letter based on their overall prescribing, but whose broad-spectrum prescribing met the national target. This was an opportunity to test directly the impact of adding a chart to a text-only social norm feedback letter.

### Aims

In these studies, we tested the following:

- (1) The impact on broad-spectrum prescribing of sending a social norm feedback letter targeting broad-spectrum prescribing compared to sending a social norm feedback letter targeting overall antibiotic prescribing
- (2) The impact on broad-spectrum prescribing of sending a social norm feedback letter targeting broad-spectrum prescribing compared to sending no letter
- (3) The impact on overall prescribing of adding a bar chart to a social norm feedback letter targeting overall prescribing that was text-only

## Methods

### Study design

We were able to send a letter from the CMO for England to GPs in practices in England. The primary aim was to investigate whether social norm feedback about broad-spectrum prescribing would be effective at reducing broad-spectrum prescribing, targeting GPs in practices that were above the 2017-2019 Quality Premium target of 10%. However, we were sensitive to whether practices were meeting the Quality Premium target for overall antibiotic prescribing; no practice that met the overall target would be sent a letter. In the 2017-2019 Quality Premium, the overall target was to be at or below 1.161 Antibacterial Items/STAR-PU [2]. (Since the amount of antibiotics prescribed by a practice is dependent on the number of patients in the practice and their demographic characteristics, the measure of overall antibiotic prescribing that is targeted is denominated in items per Specific Therapeutic Group Age-sex weightings Related Prescribing Units (STAR-PU), which is total antibiotic prescribing weighted by number and characteristics of patients registered in the practice.) The new NHS Oversight Framework, introduced in 2019-2020, further reduced the target to make it at or below 0.965 Antibacterial Items/STAR-PU [14]. It was felt that we should not write to GPs whose practices were already meeting the target, so only practices whose prescribing was greater than 0.965 Antibacterial Items/STAR-PU were sent a letter. We categorised practices whose prescribing was above the old target of 1.161 Antibacterial Items/STAR-PU as relatively high prescribing practices and those whose prescribing was between 0.965 Antibacterial Items/STAR-PU and 1.161 Antibacterial Items/STAR-PU as relatively moderate prescribers.

We sent a social norm feedback letter about broad-spectrum prescribing (for short, ‘broad-spectrum letter’) to practices with practices with broad-spectrum prescribing of greater than 10% and relatively high or relatively moderate overall prescribing. For a relatively high prescribing practice, the usual practice would be a social norm feedback letter about their overall antibiotic prescribing (for short, ‘overall prescribing letter’), whereas for a relatively moderate prescribing practice, the usual practice would be no letter. Therefore, we had two different trials aiming to reduce broad-spectrum prescribing, with different samples and different controls. For relatively high prescribing practices that met the broad-spectrum target, we compared the standard overall prescribing letter to a social norm feedback letter with a bar chart (for short, ‘overall prescribing letter with chart’).

In summary, we ran three two-armed trials:

- (1) *Broad-spectrum letter vs control letter trial*: testing whether a broad-spectrum letter would reduce the proportion of broad-spectrum prescribing compared to an overall prescribing social letter; participating practices had > 10% broad-spectrum prescribing and relatively high overall prescribing.
- (2) *Broad-spectrum letter vs no letter trial:* testing whether a broad-spectrum letter would reduce the proportion of broad-spectrum prescribing compared to no letter; participating practices had > 10% broad-spectrum prescribing and relatively moderate overall prescribing.
- (3) *Overall prescribing letter with chart vs control letter trial:* testing whether an overall prescribing letter with a chart reduced overall prescribing compared to an overall prescribing letter without a chart; participating practices had relatively high overall prescribing but their broad-spectrum prescribing was < 10%.

For each trial, we included all GP practices in England who met the eligibility requirements. We randomly allocated half to the intervention group and half to the control group. Each trial tested an intervention against the standard practice: in two trials, the standard practice was an overall prescribing letter; in the third, it was no letter. We thought that a bar chart was unlikely to reduce the effect of the letter, so we used charts in all our intervention letters, including the new broad-spectrum letters. Five of the six groups were sent a letter from the CMO and a ‘Treating your infection’ leaflet. The letters were sent through the post and timed so that they would arrive at the GP practices at the beginning of November 2018. (See the ‘Interventions’ section for more details and the ‘Additional file 1’ section for examples of the letter and the leaflet).

### Participants

GP practices were included if their rate of dispensed antibiotics was more than 1.161 Antibacterial Items/STAR-PU for the 12 months (June 2017 to May 2018), or if it was more than 0.965 Antibacterial Items/STAR-PU and more than 10% were broad-spectrum items for the twelve months. These data are collected for each GP practice (individual prescribers’ data are not available) on a monthly basis by the NHS Business Services Authority. Public Health England makes these data available on Fingertips (https://fingertips.phe.org.uk/profile/amr-local-indicators/data#page/0/gid/1938132909/pat/46/par/E39000030/ati/19/are/E38000010). For overall prescribing, we used BNF code 5.1. For broad-spectrum prescribing, we used the sum of BNF product code equal to 0501013K0 (Co-Amoxiclav), or BNF product code starts with 050102 (i.e. cephalosporins), and BNF product code starts with 050112 (i.e. Quinolones). For both overall prescribing and broad-spectrum prescribing, we divided the number of items prescribed by the Specific Therapeutic group Age-Sex Related Prescribing Units (STAR-PU), in order to get a number of items prescribed per 1000 patients in the practice population when adjusted for age and sex.

Ethical approval was obtained from the PHE Research Ethics and Governance Group (R&D 193), which was the organisation’s equivalent of a Research Ethics Committee with comparable processes where trial designs are scrutinised by research ethics experts. The Research Ethics and Governance Group waived participant consent for this trial, since obtaining consent would invalidate the results and create a burden greater than the intervention itself.

### Interventions

Letters from the Chief Medical Officer (CMO), addressed to individual GPs with the Department of Health and Social Care logo on the envelope, were posted with an anticipated delivery date at the beginning of November 2018 (we could not control exactly when they would arrive). The timing of the letter was designed to complement the ‘Keep Antibiotics Working’ campaign, which launched in October 2018, a patient-facing campaign to reduce the general public’s expectation for antibiotics and raise awareness of the risks of antibiotic resistance.

### Broad-spectrum letter vs control letter trial

#### Letter A1 (control)

The header said that ‘[practice name] prescribes more antibiotics than 80% of practices in England’. Inside the text of the letter, it said that ‘I am specifically writing to your practice because the great majority (80%) of practices in England prescribe fewer antibiotics per head (after adjustments for age and sex) than yours.’ There was no visual representation of prescribing.

#### Letter A2 (intervention)

The header said that ‘[practice name] prescribes a higher proportion of broad-spectrum antibiotics than xx% of practices in England’. Inside the text of the letter, it said that ‘I am specifically writing to your practice because the great majority (xx%) of practices in England prescribe a lower proportion of broad-spectrum antibiotics than yours.’ There was also a bar chart showing broad-spectrum prescribing compared to the practices’ peers (average broad-spectrum prescribing).

### Broad-spectrum letter vs no letter trial

#### Letter C2 (intervention)

The header said that ‘[practice name] prescribes a higher proportion of broad-spectrum antibiotics than xx% of practices in England’. Inside the text of the letter, it said that ‘I am specifically writing to your practice because the great majority (xx%) of practices in England prescribe a lower proportion of broad-spectrum antibiotics than yours.’ There was also a bar chart showing broad-spectrum prescribing compared to the practices’ peers (average broad-spectrum prescribing).

### Overall prescribing letter with chart vs control letter trial

#### Letter B1 (control)

The header said that [practice name] prescribes more antibiotics than 80% of practices in England’. Inside the text of the letter, it said that ‘I am specifically writing to your practice because the great majority (80%) of practices in England prescribe fewer antibiotics per head (after adjustments for age and sex) than yours.’ There was no visual representation of prescribing.

#### Letter B2 (intervention)

The header said that [practice name] prescribes more antibiotics than 80% of practices in England’. Inside the text of the letter, it said that ‘I am specifically writing to your practice because the great majority (80%) of practices in England prescribe fewer antibiotics per head (after adjustments for age and sex) than yours.’ There was also a bar chart showing broad-spectrum prescribing compared to the practices’ peers (average broad-spectrum prescribing).

A summary of the six trial arms and the letters they were sent is in Table 1. Note the intervention letters for the trials targeting broad-spectrum prescribing (A2 and C2) were the same. The two standard practice control letters, targeting overall prescribing (A1 and B1), were the same, and the headline message was that which had been used in previous years saying ‘the great majority (80%) of practices in England prescribe fewer antibiotics per head’. The new intervention letter was more specific, giving the exact per cent, which was always at least 80. In total, there were three different letters used in the five arms that were sent letters and a sixth arm that had no letter.

**Table 1:** Summary of the interventions in the six trial arms

**Overall prescribing in the last 12 months**	**Broad-spectrum prescribing in the last 12 months**	**Category**	**Headline message**	**Secondary message**	**Visual representation**	**Count of practices randomized into the group**
**Broad-spectrum letter vs control letter trial**						
**More than 1.161 items per STAR-PU (top 20%)**	> 10%	Letter A1 (standard practice as control, overall prescribing letter)	[Practice name] prescribes more antibiotics than 80% of practices in England.	I am specifically writing to your practice because the great majority (80%) of practices in England prescribe fewer antibiotics per head (after adjustments for age and sex) than yours.	None	201
		Letter A2 (intervention letter, broad-spectrum prescribing with chart)	[Practice name] prescribes a higher proportion of broad-spectrum antibiotics than xx% of practices in England.	I am specifically writing to your practice because the great majority (xx%) of practices in England prescribe a lower proportion of broad-spectrum antibiotics than yours.	Broad-spectrum prescribing compared to peers (average)	202
**Broad-spectrum letter vs no letter trial**						
**0.965 items per STAR-PU**	> 10%	No letter (standard practice, control).	n/a	n/a	n/a	344
		Letter C2 (intervention letter, broad-spectrum prescribing with chart)	[Practice name] prescribes a higher proportion of broad-spectrum antibiotics than xx% of practices in England.	I am specifically writing to your practice because the great majority (xx%) of practices in England prescribe a lower proportion of broad-spectrum antibiotics than yours.	Broad-spectrum prescribing compared to peers (average)	344
**Overall letter with chart vs control letter trial**						
**More than 1.161 items per STAR-PU (top 20%)**	< 10%	Letter B1 (standard practice overall prescribing letter, control)	[Practice name] prescribes more antibiotics than 80% of practices in England.	I am specifically writing to your practice because the great majority (80%) of practices in England prescribe fewer antibiotics per head (after adjustments for age and sex) than yours.	None	409
		Letter B2 (intervention letter, overall prescribing with chart)	[Practice name] prescribes more antibiotics than xx% of practices in England.	I am specifically writing to your practice because the great majority (xx%) of practices in England prescribe fewer antibiotics per head (after adjustments for age and sex) than yours.	Overall prescribing compared to peers (average)	409

All the letters listed four simple actions that the recipient could take to reduce unnecessary prescriptions of antibiotics: switching to narrow-spectrum antibiotics, giving patients advice on self-care, offering a delayed prescription, and talking about the issue with other prescribers in the practice. All letters had the advice to switch to narrow-spectrum antibiotics, even if the target in the header was overall prescribing. The action to switch from broad spectrum was new that year, but the other three actions were the same as in previous years [4, 5]. All letters were accompanied by a copy of the patient-focused ‘Treating your infection’ leaflet developed for the Treat Antibiotics Responsibly, Guidance, Education and Tools (TARGET) programme, which was also the same as in previous years.

### Outcomes and sample size

The primary outcome of the two trials targeting broad-spectrum prescribing was the proportion of broad-spectrum items prescribed adjusted for STAR-PU for the 6 months following the intervention (November 2018 to April 2019). Since a decrease in percentage broad-spectrum prescribing could be achieved either by a decrease in broad-spectrum prescribing or an increase in overall prescribing, secondary outcomes were the total amount of broad-spectrum items prescribed adjusted for STAR-PU and total broad-spectrum prescribing rates adjusted by STAR-PU, for the 6 months following the intervention (November 2018 to April 2019). This was particularly relevant in trial A, where the control letter targeted overall prescribing.

The primary outcome of the *overall letter with chart vs control letter trial* was overall antibiotic prescribing rates adjusted by STAR-PU, for the 6 months following the intervention (November 2018 to April 2019). Secondary outcomes were the total amount of broad-spectrum items prescribed adjusted for STAR-PU and total broad-spectrum prescribing rates adjusted by STAR-PU, for the 6 months following the intervention (November 2018 to April 2019).

Our sample sizes were fixed by the number of eligible practices for each trial. Prior to running the trials, we determined that, given our sample sizes, in the *broad-spectrum letter vs control letter trial* and the *broad-spectrum letter vs no control trial*, we had 80% power to detect a 2% difference in prescribing between conditions, controlling for baseline prescribing behaviour, which was considered to be a reduction that would justify writing a letter. The *Overall letter with chart vs control letter trial* was not powered to this level, but since those practices would be sent a letter anyway, it was a pragmatic opportunity to run a test.

### Randomisation and masking

We randomly assigned GP practices to the intervention or control group in each trial using a random number generator. Participants in intervention groups are likely to have been aware of the interventions they were assigned to but may have been unaware that they were involved in a trial. Since the prescribing dataset had to be matched with the dataset of GP names, it was not practical to blind the study team to group assignment.

### Statistical analysis

The monthly prescribing rates by different GP practices were viewed as independent (i.e. practice A is not correlated to practice B), while month-by-month prescribing rates from the same GP practices were viewed as potentially correlated (i.e. practice A in September correlated to practice A in October). Therefore, the study employed a statistical modelling approach that allows the monthly prescribing rates from the same GP to be correlated, by allowing the model’s residuals to be correlated.

A log transformation was applied to the outcome data after studying the residuals under an initial model. The log-transformed data were then modelled jointly for the three trials rather than separately. We used an autoregressive first-order model, AR (1), to accommodate the month-by-month dependence of the outcome measures. The variance components including correlation parameters were allowed to be varied by the trials. Some of the outliers were downweighted using the inverse of the square of the standard residual in the final models. The model estimated the treatment effect of receiving the intervention letter on monthly GP-level antibiotic prescribing in the 6 months following the intervention. The models included a time trend in addition to the intervention. Variance component estimates are also available from the final model. The data analysis was done in SAS using the [SAS/STAT] software, version 9.4 of the SAS 64 BIT WIN [15].

## Results

**Fig. 1:**
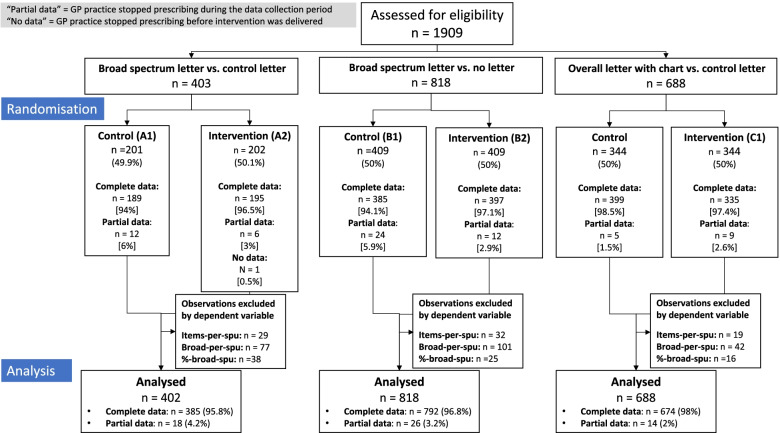
Trial profile

The *broad-spectrum letter vs control letter trial* had a sample size of 403 practices; 201 of these practices were assigned to the control group, A1, while 202 practices were in the intervention group, A2. The *broad-spectrum letter vs no letter trial* had a sample size of 688 practices, with 344 in the control group, C1, and 344 in the intervention group, C2. The *overall letter with chart vs control letter trial* had a sample size of 818 practices, 409 in the control, B1, and 409 in the intervention group, B2 (see the trial profile in Fig. 1).

### Excluded observations

We reviewed the STAR-PU values in order to determine which observations would be excluded from the main analysis. A low STAR-PU may indicate that a practice was closed or merged with another practice, with patient data gradually transitioned over to another practice. The expected value of antibiotic items per STAR-PU was around 1. To identify merged or closed practices, for each trial and each dependent variable, we identified the observations where STAR-PU was in the bottom 1% while the prescribing was above 99%. Exclusions for each variable and in each trial are given in the trial flow chart (Fig. 1).

### Broad-spectrum letter vs control letter trial

**Fig. 2:**
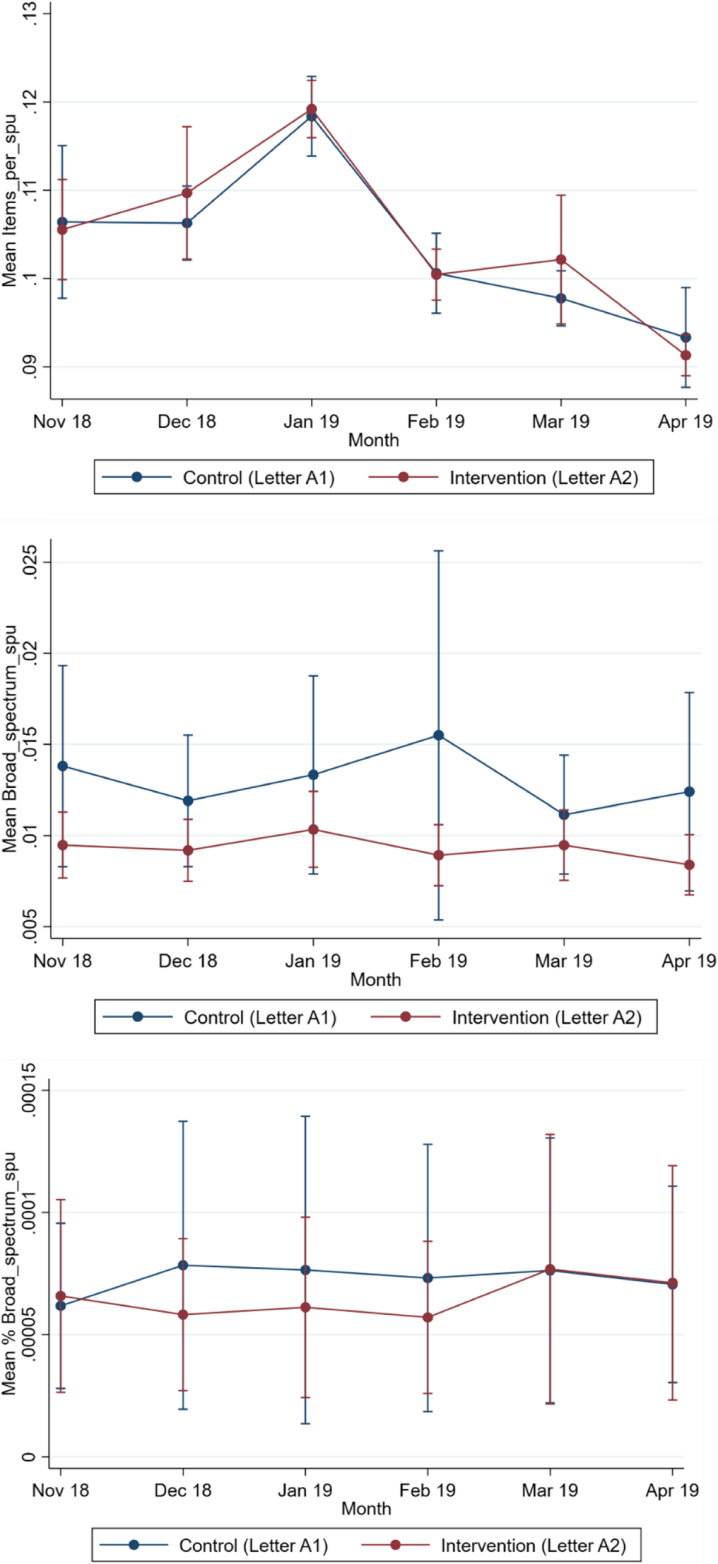
Monthly trend of prescribing means over 6 months for *broad-spectrum letter vs control letter trial*

By April 2019, there were 379 GP practices reporting the total item prescribing data and 365 GP practices reporting broad-spectrum prescribing data. The number of practices included in the analysis each month is shown in Table 2, along with the 6 months of prescribing data from November 2018 to March 2019 inclusive. The monthly mean for each arm is graphed in Fig. 2 for all three dependent variables.

**Table 2:** Antibiotic prescribing rates per STAR-PU for the intervention and control groups in the *broad-spectrum letter vs control letter trial*

	Total items per STAR-PU		Total broad spectrum per STAR-PU		% broad spectrum per STAR-PU	
	Control, M (SD) [*n*]	Intervention, M (SD) [*n*]	Control, M (SD) [*n*]	Intervention, M (SD) [*n*]	Control, M (SD) [*n*]	Intervention, M (SD) [*n*]
November 2018	0.106 (0.06) [191]	0.106 (0.04) [198]	0.014 (0.04) [185]	0.010 (0.01) [190]	0.0001 (0.0002) [183]	0.0001 (0.0003) [190]
December 2018	0.106 (0.03) [190]	0.110 (0.05) [197]	0.012(0.02) [184]	0.009 (0.01) [190]	0.0001 (0.0004) [183]	0.0001 (0.0002) [190]
January 2019	0.118 (0.03) [190]	0.119 (0.02) [196]	0.013 (0.04) [185]	0.010 (0.01) [189]	0.0001 (0.0004) [183]	0.0001 (0.0003) [189]
February 2019	0.101 (0.03) [189]	0.100 (0.02) [195]	0.016 (0.07) [182]	0.009 (0.01) [188]	0.0001 (0.0004) [181]	0.0001 (0.0002) [188]
March 2019	0.098 (0.02) [188]	0.102 (0.05) [196]	0.011 (0.02) [180]	0.010 (0.01) [187]	0.0001 (0.0004) [180]	0.0001 (0.0004) [187]
April 2019	0.093(0.04) [186]	0.091 (0.02) [193]	0.012 (0.04) [179]	0.008 (0.01) [186]	0.0001 (0.0003) [179]	0.0001 (0.0003) [185]

Number of practices with data for each dependent variable and month are shown in square brackets

There was no evidence of an effect of the intervention for percentage broad-spectrum prescribing or total broad-spectrum prescribing. However, there was higher overall prescribing in the intervention group, *β* = .023, *z* = 2.04, *p* = .041 (see Table 3).

**Table 3:** AR(1) models of prescribing for *broad-spectrum letter vs control letter trial*

*Parameter*	*Est*	*SE*	*z-value*	*p-value*
***Total antibiotic prescribing per STAR-PU***				
Intercept	− 2.253	.01	− 200.81	< .0001
Sent intervention letter	.023	.01	2.04	.041
Trend	− .022	.00	− 9.94	< .0001
***Total broad-spectrum prescribing per STAR-PU***				
Intercept	− 5.224	.07	− 70.75	< .0001
Sent intervention letter	.132	.10	1.38	.17
Trend	− .0231	.01	− 2.67	.0076
***Percentage broad-spectrum prescribing per STAR-PU***				
Intercept	− 11.071	.10	− 116.26	< .0001
Sent intervention letter	− .199	.13	− 1.51	.13
Trend	− .004	.01	− .043	.67

The model showed a statistically significant negative trend in total antibiotic prescribing, *β* = − .022, *z* = − 9.94, *p* < .0001, but no statistically significant trend in total broad-spectrum prescribing or percentage broad-spectrum prescribing (see Table 3).

### Broad-spectrum letter with chart vs no letter trial

**Fig. 3:**
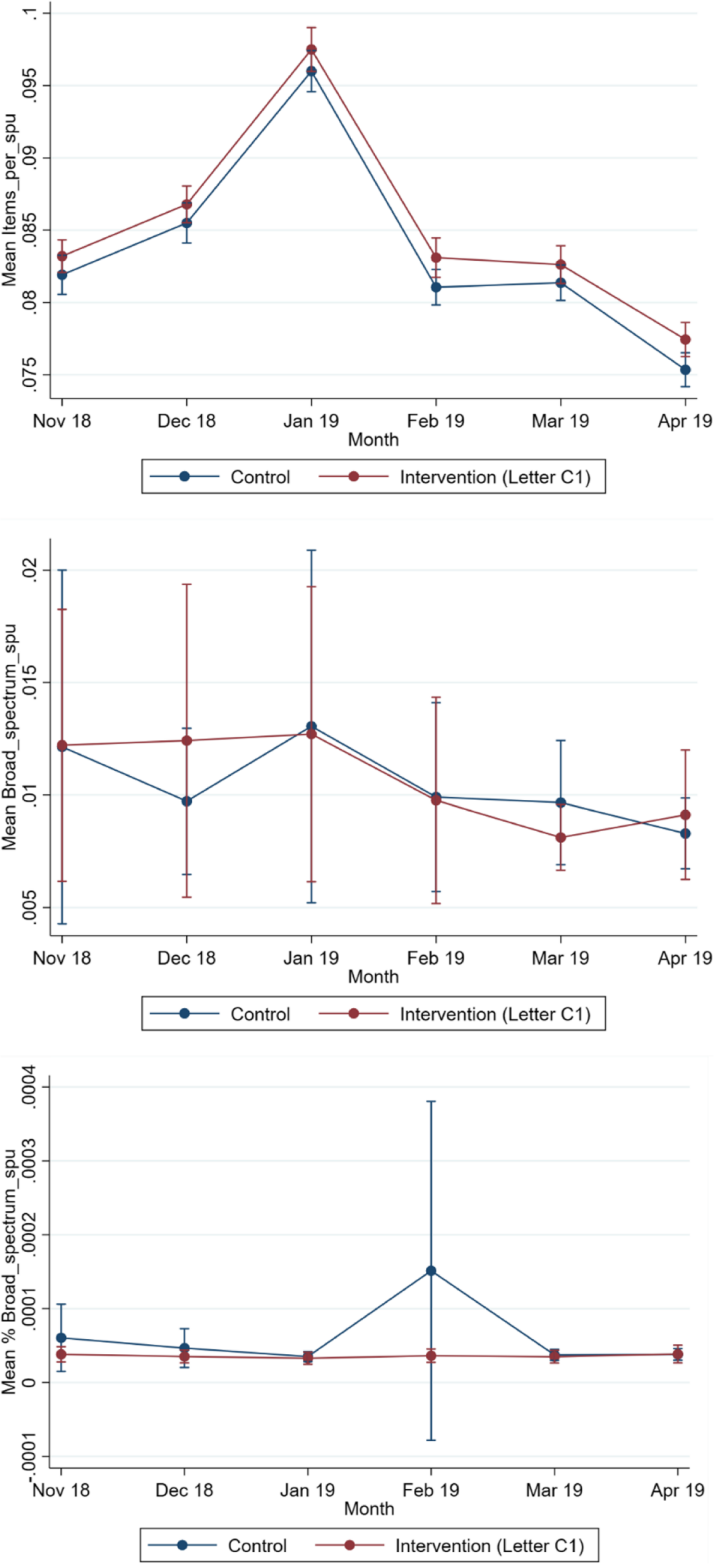
Monthly trend of prescribing mean over 6 months for *broad-spectrum letter with chart vs no letter trial*

By April 2019, there were 672 GP practices reporting total item prescribing data and 631 GP practices reporting broad-spectrum prescribing data. The number of practices included in the analysis each month is shown in Table 4, along with the 6 months of prescribing data from November 2018 to March 2019 inclusive. The monthly mean for each arm is graphed in Fig. 3 for all three outcome measures.

**Table 4:** Antibiotic prescribing rates per STAR-PU for the intervention and control groups in the *broad-spectrum letter with chart vs no letter trial*

	Total items per STAR-PU		Total broad spectrum per STAR-PU		% broad spectrum per STAR-PU	
	Control, M (SD) [*n*]	Intervention, M (SD) [*n*]	Control, M (SD) [*n*]	Intervention, M (SD) [*n*]	Control, M (SD) [*n*]	Intervention, M (SD) [*n*]
November 2018	0.082 (0.01) [338]	0.083 (0.01) [339]	0.012 (0.07) [313]	0.012 (0.06) [332]	0.0001 (0.0004) [312]	0.0000 (0.0001) [330]
December 2018	0.086 (0.01) [337]	0.087 (0.01) [339]	0.010 (0.02) [311]	0.012 (0.06) [331]	0.0000 (0.0002) [309]	0.0000 (0.0001) [329]
January 2019	0.096 (0.01) [336]	0.098 (0.01) [339]	0.013 (0.07) [309]	0.013 (0.06) [330]	0.0000 (0.0001) [306]	0.0000 (0.0001) [328]
February 2019	0.081 (0.01) [336]	0.083 (0.01) [339]	0.010 (0.04) [306]	0.010 (0.04) [328]	0.0002 (0.0020) [304]	0.0000 (0.0001) [327]
March 2019	0.081 (0.01) [335]	0.083 (0.01) [339]	0.010 (0.02) [306]	0.010 (0.01) [327]	0.0000 (0.0001) [303]	0.0000 (0.0001) [326]
April 2019	0.075 (0.01) [334]	0.077 (0.01) [338]	0.010 (0.01) [305]	0.009 (0.03) [326]	0.0000 (0.0001) [302]	0.0000 (0.0001) [324]

Number of practices with data for each outcome measure and month are shown in square brackets

There was no evidence of an effect of the intervention for total broad-spectrum prescribing or percentage broad-spectrum prescribing (see Table 5). However, the intervention group had lower total antibiotic prescribing, *β* = − .016, *z* = − 2.45, *p* = .0143. Total antibiotic prescribing decreased over time, *β* = − .018, *z* = − 12.23, *p* < .001, as did total broad-spectrum prescribing, *β* = − .026, *z* = − 4.02, *p* < .001. However, there was no statistically significant time trend for percentage broad-spectrum prescribing (see Table 5).

**Table 5:** AR(1) model for *broad-spectrum letter with chart vs no letter trial*

*Parameter*	*Est*	*SE*	*z-value*	*p-value*
***Total antibiotic prescribing per STAR-PU***				
Intercept	− 2.422	.01	− 355.44	< .001
Sent intervention letter	− .016	.01	− 2.45	.014
Trend	− .018	.00	− 12.23	< .001
***Total broad-spectrum prescribing per STAR-PU***				
Intercept	− 5.363	.05	− 101.21	< .001
Sent intervention letter	.050	.07	.72	.47
Trend	− .026	.01	− 4.02	< .001
***Percentage broad-spectrum prescribing per STAR-PU***				
Intercept	− 11.271	.07	− 153.14	< .001
Sent intervention letter	.006	.10	.06	.95
Trend	− .011	.01	− 1.5	.13

#### Overall letter with chart vs control letter trial

**Fig. 4:**
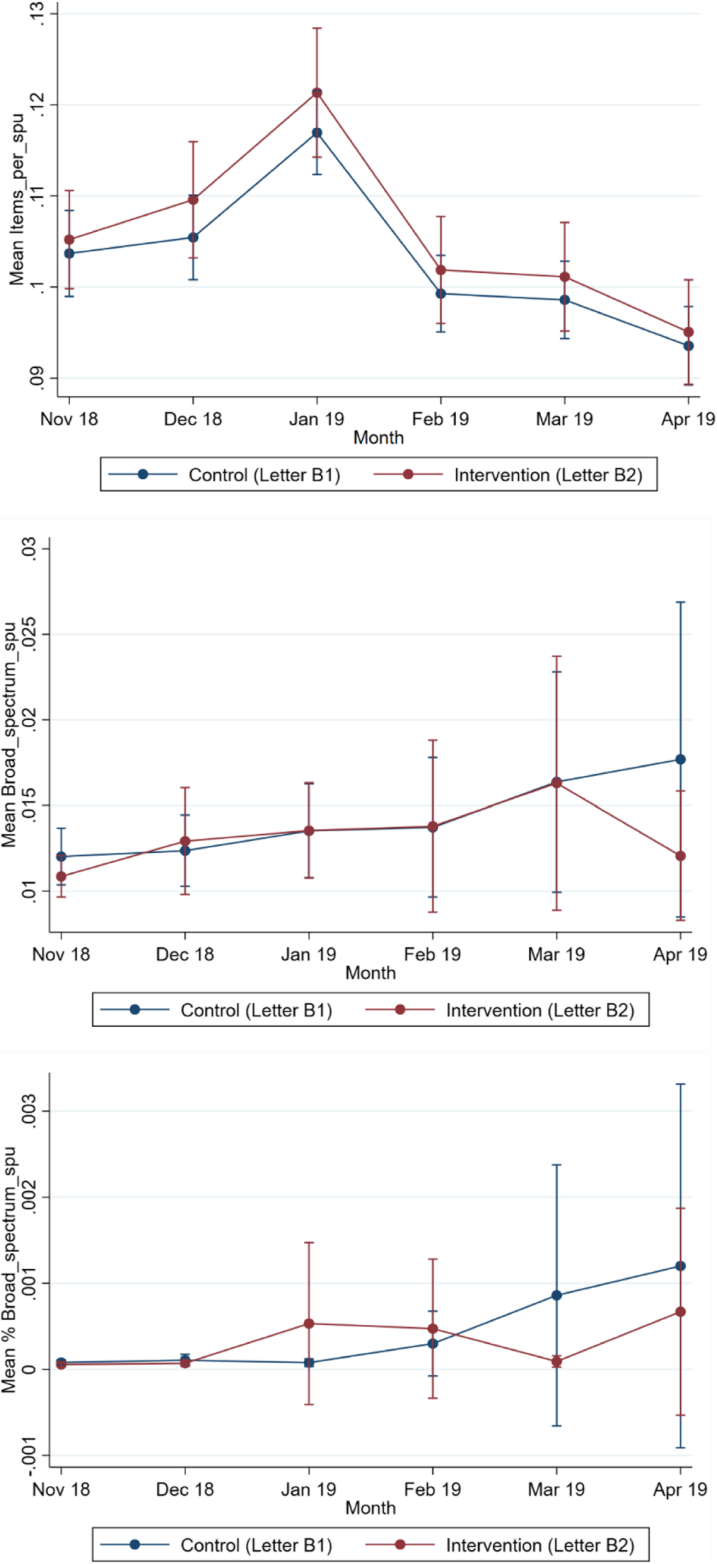
Monthly trend of prescribing means over 6 months for *overall letter with chart vs control letter trial*

By April 2019, there were 788 GP practices reporting total item prescribing data and 745 GP practices reporting broad-spectrum prescribing data. The number of practices included in the analysis each month is shown in Table 6, along with the 6 months of prescribing data from November 2018 to March 2019 inclusive. The monthly mean for each arm is graphed in Fig. 4 for all three dependent variables.

**Table 6:** Antibiotic prescribing rates per STAR-PU for the intervention and control groups in the *overall letter with chart vs control letter trial*

	**Total items per STAR-PU**		**Total broad spectrum per STAR-PU**		**% broad spectrum per STAR-PU**	
	Control, M (SD) [*n*]	Intervention, M (SD) [*n*]	Control, M (SD) [*n*]	Intervention, M (SD) [*n*]	Control, M (SD) [*n*]	Intervention, M (SD) [*n*]
**November 2018**	0.104 (0.05) [397]	0.105 (0.05) [398]	0.012 (0.02) [388]	0.011 (0.01) [370]	0.0001 (0.0003) [387]	0.0001 (0.0002) [369]
**December 2018**	0.105 (0.05) [395]	0.110 (0.06) [398]	0.012 (0.02) [385]	0.013 (0.03) [368]	0.0001 (0.0007) [385]	0.0001 (0.0003) [367]
**January 2019**	0.117 (0.05) [394]	0.121 (0.07) [397]	0.014 (0.03) [387]	0.014 (0.03) [367]	0.0001 (0.0004) [385]	0.0005 (0.0091) [366]
**February 2019**	0.099 (0.04) [393]	0.102 (0.06) [396]	0.014 (0.04) [385]	0.014 (0.05) [366]	0.0003 (0.0038) [385]	0.0005 (0.0079) [365]
**March 2019**	0.099 (0.04) [393]	0.101 (0.06) [395]	0.016 (0.06) [384]	0.016 (0.07) [365]	0.0009 (0.0151) [383]	0.0001 (0.0006) [363]
**April 2019**	0.094 (0.04) [392]	0.095 (0.06) [395]	0.018 (0.09) [383]	0.012 (0.04) [362]	0.0012 (0.0210) [383]	0.0007 (0.0116) [360]

Number of practices with data for each outcome measure and month are shown in square brackets

There was no evidence of an effect of the intervention on total antibiotic prescribing, total broad-spectrum prescribing, and percentage broad-spectrum prescribing (see Table 7). There was a decrease in total antibiotic prescribing over time, *β* = .022, *z* = − 13.58, *p* < .001, and in total broad-spectrum prescribing, *β* = − .022, *z* = − 3.68, *p* <.001, but there was no statistically significant time trend for percentage broad-spectrum prescribing (see Table 7).

**Table 7:** AR(1) models for *overall letter with chart vs control letter trial*

*Parameter*	*Est*	*SE*	*z-value*	*p-value*
***Total antibiotic prescribing per STAR-PU***				
Intercept	− 3.367	.01	− 274.56	< .001
Sent intervention letter	− .002	.01	− .24	.81
Trend	− .022	.00	− 13.58	< .001
***Total broad-spectrum prescribing per STAR-PU***				
Intercept	− 5.103	.05	− 100.61	< .001
Sent intervention letter	.054	.07	.8	.42
Trend	− .022	.01	− 3.68	< .001
***Percentage broad-spectrum prescribing per STAR-PU***				
Intercept	− 10.951	.07	− 148.97	< .001
Sent intervention letter	− .106	.10	− 1.08	.28
Trend	− .005	.01	− .76	.45

## Discussion

Social norm feedback about a practice’s relatively high percentage of broad-spectrum prescribing did not reduce the proportion of broad-spectrum antibiotics prescribed, either compared to a social norm feedback letter targeting overall prescribing or compared to no letter. There was a decrease in the overall levels of antibiotic prescribing over time across all groups and a decrease in absolute broad-spectrum prescribing over all groups. We found no additional effect of the letters on broad-spectrum prescribing. However, in the *broad-spectrum letter vs control letter trial*, the intervention group had higher overall antibiotic prescribing, which may not be surprising because the control letter targeted overall prescribing, whereas the intervention letter only targeted broad-spectrum prescribing. So, this could be seen as a success of the control letter.

Previous international evidence has shown that social norm feedback to high prescribers of antibiotics can decrease prescribing [4, 5, 8–10]. However, our attempt to use this method to decrease the proportion of broad-spectrum antibiotics prescribed was not successful. Nor was a previous trial run in February 2018 that sent social norm feedback to the minority of practices whose prescribing was increasing [16]. The trial we report here had greater specificity in target behaviour (broad-spectrum prescribing), and the February 2018 trial had greater specificity in the audience (practices whose prescribing was increasing), which should theoretically have improved the impact. However, unlike the large body of international evidence, in our *broad-spectrum letter vs control letter trial*, there was an active control group. If the control group was already reducing prescribing, then the intervention would have needed to be even more impactful in order to find a difference, so potentially there was a floor effect.

Including a chart in a social norm feedback letter about overall prescribing did not have an effect on prescribing compared to a social norm feedback letter without a chart. This contrasts with the findings of the Australian team, on whose designs we based our charts, who found a striking 12.3% decrease in prescribing compared to a no-letter control [8]. We used the same bar chart as the Australian letter because it had been used in a successful trial and because the design with the pills seemed visually appealing, which may help comprehension of graphs [17, 18]. Bar charts have also been used in other successful social norm feedback trials [19–21]. However, one might wonder whether other ways of visualising the data would have had more impact. For instance, we could have used line graphs or pie charts or a pictogram. There is limited evidence on how different types of visualisations affect understanding; their effects will also depend on the numeracy of the viewer and their familiarity with the type of information being conveyed [22–26]. However, for simple comparisons, chart type may not make a difference [27]; bar charts are a good vehicle for making comparisons between the heights of different bars [28–31].

Our trial that tested the effect of adding a chart was not powered to find what colleagues considered to be the smallest effect size of interest due to the charts (2%). We were limited by the number of practices with prescribing in the eligible range. So, we cannot be certain that the chart did not have a small effect. The Australian team also tested some letters without charts, finding that a social norm letter with education decreased prescribing by 9.3% compared to the control and a letter with social norm feedback that promoted delayed prescribing led to a reduction of 10.4% [8]. It is tempting to infer that their charts may have had a small effect, since these are numerically smaller reductions than the 12.3% achieved with the chart; however, we note that they did not conduct statistical comparisons between letter types. Therefore, no firm conclusions about the effectiveness of adding charts can be drawn at this time.

The difference between our results and those of the Australian trial is striking. There are two key differences between the trials.

The first is the nature of the control condition. This was the fourth time that letters had been sent in England, compared to the first in Australia, so the standard practice for the control group in each country was different. The Australian control group were not sent a letter, whereas, in our trial that investigated the charts, the English control group of relatively high prescribers were sent a social norm feedback letter that had been successful in the past [4, 5]. Our use of an active control may have led to a floor effect. Further, there were downsides to adding a bar chart to our standard letter. The standard letter was only one side, but the intervention letter with a chart was two-sided. Although we put all the important information on the first side, the longer letter may have led to GPs paying less attention.

A second important difference between England and Australia is that, in Australia, they collect data on individual GP prescribing, so their GPs received feedback on their own prescribing, whereas in the UK, prescribing data is only available at the level of the practice, so GPs in our trial received feedback on the level of prescribing in their practice. When told about high practice levels of prescribing, GPs often ascribe the high prescribing rate to other GPs in the practice [1]. If GPs are given data about their own prescribing, then the prescribing is clearly due to them and they may be more likely to take action. That might lead to a generally larger effect on the Australian letters.

Potentially, the social norm feedback intervention would be more effective if prescribing measures were more personalised for each GP (in comparison to the practice-level data that we had access to). The original UK social norm feedback trial used localised feedback, comparing prescribing to other practices in the local area team [2], but this could not be repeated because local area teams were abolished. A qualitative study of social norm feedback letters found that GPs and other primary care prescribers wanted tailored and localised data with peer-to-peer comparisons [32]. An analysis of primary care electronic health records, which assessed **e**ight different measures of antibiotic prescribing (including overall and incidental antibiotic prescribing, repeat antibiotic courses and extent of risk-based prescribing for hospital admissions), found that there was considerable variability between the various antibiotic measures for individual clinicians but weak correlation coefficients for most measures [33]. The majority of clinicians (95.8%) prescribed at least one antibiotic measure in a way that was above the medians of their peers. Delivering individual feedback on specific prescribing measures may be the best way to enhance the English social norm feedback letters’ effectiveness. Given the variety of prescribing measures being fed back, it might be less resource-intensive to deliver feedback digitally. It is possible to extract prescribing data and use a dashboard infrastructure to display summary data and recommendations to individual practices or clinicians, combining advanced data analytics with tailored feedback [3]. This may offer a way forwards.

## Conclusion

A social norm feedback letter informing general practitioners that their broad-spectrum prescribing is above their peers was not effective at reducing the percentage of broad-spectrum prescribing. Adding a chart to the standard practice active control letter, aimed at overall prescribing, did not make this social norms intervention more effective although caution should be used in interpreting this result as the study was underpowered.

## Supplementary Information

**Additional file 1.** 

## Data Availability

The data in the paper is all publicly available: https://fingertips.phe.org.uk/profile/amr-local-indicators/data#page/0/gid/1938132909/pat/46/par/E39000030/ati/19/are/E38000010.

## Abbreviations

AMR: Antimicrobial resistance
BNF: British National Formulary
CMO: Chief Medical Officer
GP: General practitioner
PHE: Public Health England
RCT: Randomized controlled trial
STAR-PU: Specific Therapeutic Group Age-sex weightings Related Prescribing Units
