# Supplementary material for: Using text and charts to provide social norm feedback to general practices with high overall and high broad-spectrum antibiotic prescribing: a series of national randomised controlled trials
Source: Trials. 2022 Jun 18;23:511. doi: 10.1186/s13063-022-06373-y (PMC9206287; doi:10.1186/s13063-022-06373-y)
Supplement: Supplementary file 1 — Additional file 1. [file 13063_2022_6373_MOESM1_ESM.zip › Appendix 2_Letter A2 & C2_Intervention LetterR2.docx]

[GP_Name]
[Address 1]
[Address 2], [Address 3]
[Address 4], [Address 5]

October 2018

[Practice NAME] prescribes a higher proportion of broad spectrum antiobiotics than xx% of practices in England

**1. SWITCH to** narrow spectrum instead of broad spectrum antibiotics, where appropriate. Broad spectrum antibiotic prescribing is a significant driver of resistance.

**2. TELL patients about their personal risks.** Discuss side effects (allergic reactions, gastrointestinal conditions) and personal resistance, which may last for up to twelve months after a course of antibiotics (Costelloe et al, BMJ. 2010).

**3. SUBSTITUTE a prescription for the enclosed TARGET leaflet giving the patient self-care advice to take home with them.** A printable version is available at: [www.rcgp.org.uk/targetantibiotics](http://www.rcgp.org.uk/targetantibiotics)

**4. Offer a DELAYED** prescription.
This results in fewer patients using antibiotics and is associated with similar symptomatic outcomes to immediate prescription.
(Little et al., BMJ. 2014).

Dear Dr [GP_Surname]

**Antibiotic usage in [practice name]**

Antimicrobial resistance is already causing deaths; a patient in the USA died last year with a Klebsiella infection that was resistant to all available antimicrobial drugs.^[1]^ We have seen cases of highly resistant gram negative bacteria in the UK. Broad spectrum antibiotic prescribing is a significant driver of resistance.

**I am specifically writing to your practice because the great majority (xx%) of practices in England prescribe a lower proportion of broad spectrum antibiotics than yours.^[2]^**

You prescribed the following antibiotics between 1^st^ June 2017 and 30^th^ May 2018, compared to your peers.^[3]^

[SPACE FOR GRAPH]

I know that general practitioners are doing a demanding job in difficult circumstances and have to deal with patient pressures so, as well as running our national ‘Keep Antibiotics Working’ campaign to educate the public, I have made some suggestions for dealing with these pressures in the boxes to the right.

It is the actions of individual prescribers that make up the national picture on antimicrobial resistance. Together, these small changes can have a big effect on safeguarding your patients and antibiotics for future generations.

Your local CCG prescribing advisor can also help your practice reduce antibiotic and broad spectrum antibiotic prescribing. Links to further information are provided below.

Thank you for working with me on this.

Kind regards

**PROFESSOR DAME SALLY DAVIES**

Chief Medical Officer

1. BMJ 2017;356:j254
2. Your practice’s prescribing data are available online at https://fingertips.phe.org.uk/profile/amr-local-indicators/data. Note that practice demographics are accounted for. For more information on antimicrobial resistance, see http://antibioticguardian.com/. Patients can be referred to the “Keep Antibiotics Working” film: <http://bit.ly/2xZZ6IY>
3. The data for “Your Peers” refers to the average prescribing in England.
